# MOTH: Memory-Efficient On-the-Fly Tiling of Histological Image Annotations Using QuPath

**DOI:** 10.3390/jimaging10110292

**Published:** 2024-11-15

**Authors:** Thomas Kauer, Jannik Sehring, Kai Schmid, Marek Bartkuhn, Benedikt Wiebach, Slaven Crnkovic, Grazyna Kwapiszewska, Till Acker, Daniel Amsel

**Affiliations:** 1 Institute of Neuropathology, Justus-Liebig-University Giessen, Arndtstr. 16, 35392 Giessen, Germany; thomas.kauer@ges.thm.de (T.K.); jannik.sehring@patho.med.uni-giessen.de (J.S.); kai.schmid@patho.med.uni-giessen.de (K.S.); till.acker@patho.med.uni-giessen.de (T.A.); 2 Institute for Lung Health, Justus-Liebig University Giessen, Aulweg 128, 35392 Giessen, Germany; marek.bartkuhn@gen.bio.uni-giessen.de (M.B.); benedikt.wiebach@bioinfsys.uni-giessen.de (B.W.); slaven.crnkovic@innere.med.uni-giessen.de (S.C.); grazyna.kwapiszewska-marsh@medunigraz.at (G.K.); 3 Biomedical Informatics and Systems Medicine, Justus-Liebig-University Giessen, Aulweg 128, 35392 Giessen, Germany; 4 Medical University of Graz, Neue Stiftingtalstraße 6, 8010 Graz, Austria

**Keywords:** whole slide image, qupath, artificial intelligence, segmentation, digital pathology

## Abstract

The emerging usage of digitalized histopathological images is leading to a novel possibility for data analysis. With the help of artificial intelligence algorithms, it is now possible to detect certain structures and morphological features on whole slide images automatically. This enables algorithms to count, measure, or evaluate those areas when trained properly. To achieve suitable training, datasets must be annotated and curated by users in programs like QuPath. The extraction of this data for artificial intelligence algorithms is still rather tedious and needs to be saved on a local hard drive. We developed a toolkit for integration into existing pipelines and tools, like U-net, for the on-the-fly extraction of annotation tiles from existing QuPath projects. The tiles can be directly used as input for artificial intelligence algorithms, and the results are directly transferred back to QuPath for visual inspection. With the toolkit, we created a convenient way to incorporate QuPath into existing AI workflows.

## 1. Introduction

In histopathological image analysis, the high-throughput digitalization of tissues has led to the development of advanced computational techniques, particularly those driven by artificial intelligence (AI) algorithms [1,2]. The automated detection of structures and morphological features within digitized histopathological images, or whole slide images (WSIs), holds immense promise, affording pathologists and researchers the ability to perform automated quantification, measurement, and evaluation, provided that these algorithms are adequately trained in advance. Many approaches utilize segmentation tasks, for example to derive grading between healthy and cancerous tissues, like in colon biopsies and polyps [3].

For training, careful annotation and curation of large datasets is necessary. This task is often executed within specialized software platforms, such as QuPath [4], and saved as geometric figures, such as polygons. QuPath is one of the leading tools for the annotation of whole slide images [5], besides tools like SlideRunner [6] and HistomicsUI, which are part of the Digital Slide Archive (DSA) [7].

Analyzing WSIs presents additional challenges due to their gigapixel size. Most methods require dividing the image into smaller, more manageable patches, the so-called tiling process, where the WSI is disassembled into regions and image tiles of reasonable size (e.g., 512 × 512 px) and are extracted at areas of interest. For segmentation using machine learning (ML), usually a second image, serving as the annotation mask, with discretized values for the annotated classes, needs to be stored to be able to train the ML model properly. In current workflows, this is usually achieved by performing pre-tiling in QuPath with the built-in Groovy-based script editor and storing pairs of image tiles and masks on the hard disk. This approach generates a large amount of data pairs occupying the storage, which may make it necessary to utilize a hard disk drive (HDD) instead of a faster storage type, like solid-state-drives (SSD), with its drawbacks in request time. The pre-tiling also counteracts the flexibility of requested image regions, which may be important in performing tasks such as image augmentation or extracting overlapping tiles.

Currently, there is also no possibility to import segmentation results on this tile-level back into QuPath without additional processing steps, restricting the visual inspection to the individual tile level instead of a global overview within the original WSI.

**Figure 1:**
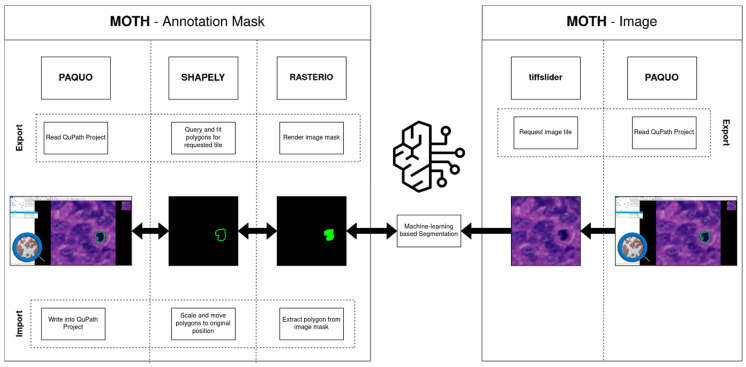
MOTH overview. MOTH is a suite of tools that facilitates the import and export of annotations and images from and into QuPath. The system is capable of establishing a connection to local AI-based algorithms.

To solve these problems, we introduce an efficient, flexible, and memory-conscious solution within the domain of histopathological image analysis: MOTH (Memory-efficient on-the-fly tiling of histopathological image annotations using QuPath). MOTH represents a transformative toolkit engineered to seamlessly integrate with existing workflows and tools, to enable the dynamic extraction of annotation tiles directly from QuPath projects during the training and prediction process. This tool provides a solution to many of the problems present when performing segmentation on histopathological image data (Figure 1). Utilizing paquo [8], we can extract annotation polygons on-the-fly and discretize them to an image mask, image tiles are extracted using tiffslide [9]. We also implemented the way back into QuPath, generating polygons out of image masks and joining adjacent polygons. In summary, MOTH provides the following functions:

- Memory efficiency and flexibility, allowing for on-the-fly requests of image tile and annotation pairs
- Global visualization of segmentation results in QuPath, recombining segmentation results on tile-level into joined polygons
- Bridging the gap between annotation tools and machine learning algorithms, allowing for seamless integration

This allows for easy utilization of annotated data in machine learning models with maximal flexibility, as well as the inspection of results in a global manner on the original data.

### Related Works

Existing tools like HistomicsTk as a part of the DSA and the wsiprocess package present the need for (on-the-fly) tiling of WSIs and annotations in Python. To process on-the-fly tiling in the DSA environment, HistomicsTK offers Python utils to extract annotation masks from a DSA slide object (https://digitalslidearchive.github.io/HistomicsTK/examples/annotations_to_semantic_segmentation_masks.html#Generate-mask-image-from-user-defined-coordinates, accessed on 14 November 2024). Besides the export of annotation masks, annotation masks can also be converted back to *DSA* slide annotations and merged to resolve tile borders (https://digitalslidearchive.github.io/HistomicsTK/examples/segmentation_masks_to_annotations.html, https://digitalslidearchive.github.io/HistomicsTK/examples/polygon_merger_from_tiled_masks.html, accessed 14 November 2024).

To export annotation masks from the tools ASAP, SlideRunner, NDP.View2, and QuPath, the general-purpose Python package wsiprocess can be used.

To create annotation masks from QuPath, the user must export annotations in a json file and manage WSIs and corresponding annotation files themselves. Wsiprocess focuses on the export of annotation masks and offers utils to directly create a pytorch dataset after patching tiles. The created pytorch datasets then utilize the patched and disk-saved masks. Utils to import annotation masks are currently not implemented in the wsiprocess module.

## 2. Material and Methods

The presented MOTH Python package enables machine learning architects to integrate QuPath annotation data directly into their workflows. This is achieved by extending the capabilities of paquo. Paquo is a Python package that enables the integration of popular Python libraries, within QuPath, a platform that traditionally supports only Groovy scripts. MOTH primarily enhances paquo by providing functions to extract tiles and their annotations in the discretized pixel space format, mostly needed in machine-learning-driven segmentation approaches. It also allows the conversion of resulting predictions back to polygons, stitching them together and enabling visualization of the predictions in QuPath, suitable for a global inspection of segmentation results. To achieve the tasks, shapely [10] and rasterio [11] are utilized. Shapely, as a spatial Python library using functions from the GEOS library [12], is used by paquo and MOTH to interact with geometric objects. Rasterio is used to read and write geospatial raster data. Within MOTH, rasterio handles the writing and reading of geometries to and from masks.

### 2.1. MOTH–Tile Export

The first set of functions, used to extract tiles and their respective annotations, aims to provide an alternative to the QuPath scripting approach. As previously mentioned, QuPath uses Groovy scripts to export annotations as shapes or images. Scripts can either be run in the interactive code editor or through a headless QuPath call.

Like QuPath, MOTH offers the export of annotated tiles. Either as a multi-class annotation image with channels for each class, or as a single-labeled image that contains a label for each pixel. For purposes where the annotations of a tile will be needed as shapely geometries, the user can call the get_tile_annot method to retrieve the annotations in the tiles area as pairs of geometries and their corresponding label.

To export the annotations of a tile, either as a mask or polygons, all the annotations of the image, provided by paquo, are used to build a STRTree as an efficient spatial search structure. The STRTree is then used to query all annotations intersecting the requested region. The intersecting parts of the polygons can be returned as polygons or rendered to a mask containing the discretized annotations of the region.

### 2.2. MOTH–Tile Import

Annotations from annotation masks can be imported into QuPath by MOTH’s save_mask_annotations function. To accomplish this, the annotations of the mask get extracted and rescaled. Afterward, the extracted annotations get translated to the correct location and will be saved in the specified image.

The import of many (small) tiles can lead to adjacent polygons of the same class being derived from different tiles. To join these adjacent tiles, MOTH additionally provides an annotation merge function to detect and merge nearby annotations. Each annotation of the image is compared with its surrounding annotations. A new annotation is created from the union of touching annotations detected as having the same class. The annotations used for the union annotation are then deleted.

Overall, MOTH provides utils to accomplish the export and import of tiles and their annotations directly in Python and on-the-fly without the need for hard disk storage between export and usage.

## 3. Results and Discussion

To demonstrate the benefits of MOTH, we evaluate it in terms of the quality of annotations and the export speed of MOTH.

The quality test will demonstrate that MOTH’s discretization contains a minimal error compared with its the Groovy script counterpart. The speed evaluation is important as the loading of images, even if performed on-the-fly, should not limit the whole training process as a bottleneck.

### 3.1. Quality of Annotations

**Figure 2:**
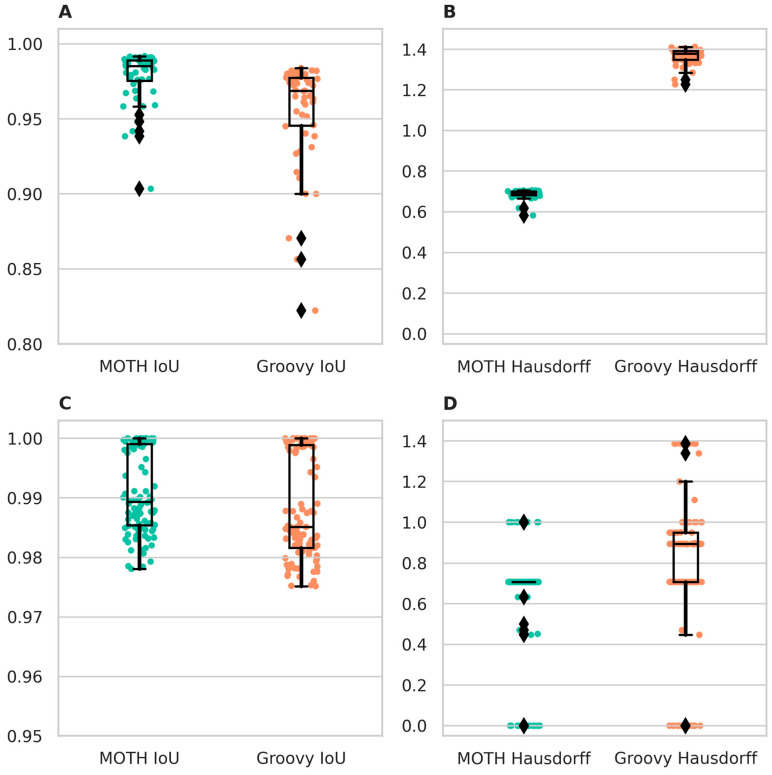
(**A**,**B**) IoU and HD of exported shapes rendered with MOTH and Groovy in the artificial dataset. (**C**,**D**) IoU and HD of exported shapes rendered with MOTH and Groovy in the mitosis dataset. Groovy results are marked in orange and MOTH results are marked in green. Diamonds represent outliers.

To demonstrate its minimal discretization error, the quality of the export performed by MOTH was compared to the QuPath built-in Groovy export (Figure 2). To compare these two export methods, different datasets were used to evaluate the performance of the export methods. The datasets were from two different kinds of data categories, one containing artificial and the other containing real-world examples. The artificial dataset contains contained 55 individual annotations. Annotations were created from a set of points which were sampled with random angles and around a fixed center. The mean distance from the points increases increased gradually with each polygon. This approach generated a dataset of polygons varying in size and shape, capturing a broad variance of possible real-world annotations. The real-world dataset contains contained 96 annotations of detected mitoses as a (neuro-)pathology example. The QuPath projects of the datasets were exported in regions with Groovy and MOTH and evaluated, analogously to Kurmi et al. 2020 [13], with the intersection over union (IoU) and Hausdorff distance (HD) between the original shapes, with subpixel accuracy, and the exported masks. The resultsare shown in (Table 1 and, Figure 2). The IoU scaling has a value between zero and one (one representing a perfect fit), and the HD measures the maximal distance between the outlines in pixels (zero for identical outlines).

The results displayed in Figure 2 and Table 1 show that MOTH exhibited superior performance compared to Groovy. The difference is more visible with the artificial dataset, as the associated polygons were smaller on average and were less smooth, which especially accounted for the differences in HD. With the real-world dataset, while we also observed better performance using MOTH, the difference was not as clear. This was due to smoother outlines and larger areas in the dataset, both of which impacted the metrics used.

To investigate quality differences between MOTH and Groovy, a QuPath project containing small defined shapes was created. We added a square that could be exactly discretized into pixels. The other shapes had pixel offsets, which means they did not align perfectly with the pixels, or were circles with and without additional offsets concerning their center. These toy examples allowed a focus of the evaluation on especially challenging cases for discretization, with subpixel offsets.

The shapes of the created QuPath project were exported with Groovy and MOTH to be compared against their ground truth polygons.

To compare the discretized shapes from both tools to their ground truth polygons, we visualized them with matplotlib and computed the IoU and HD.

**Figure 3:**
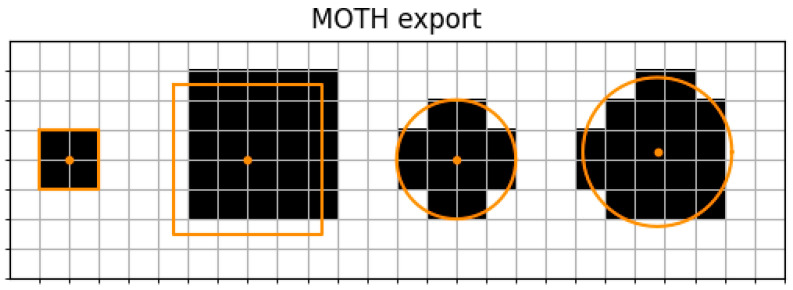
MOTH export of small shapes with pixel offsets. The figure shows the export of small ground truth shapes. The ground truth shapes are drawn as orange lines and the center of the shape is marked by an orange dot. Black areas are pixels set in the MOTH export. A high overlap with the ground truth shapes can be observed.

**Figure 4:**
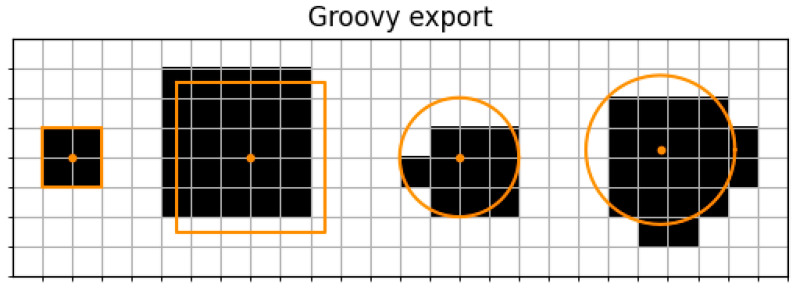
Groovy export of small shapes with pixel offsets. The figure shows the export of small ground truth shapes. In comparison to the previous figure, a lower overlap between the ground truth and the export is visible.

By visual inspection, one can observe that the simple square that aligned perfectly with the pixels was equally well treated by both tools. The larger square with the pixel offset visualizes a difference between Groovy and MOTH when it comes to the selection of representative pixels. When comparing the different circles, we observed a clear advantage of MOTH, compared to Groovy, as the selected pixels seem to represent a better filling of the geometry (Figure 3 and Figure 4).

By computing the IoU, we observed that in comparison to Groovy, MOTH provided a higher IoU on circles, with values of 0.852 compared to 0.6693 from Groovy for the first occurring circle, and 0.8218 compared to 0.6487 from Groovy for the second occurring circle.

On rectangles, MOTH and groovy Groovy yielded identical IoUs, namely, 1.0 on the left square and 0.6807 on the right square (Table 2).

### 3.2. Speed

As in machine learning approaches, especially deep learning, it is necessary to provide image masks multiple times to the model. It is essential that the loading process of the masks is sufficiently fast and does not serve as a bottleneck. Therefore, we investigated the time it takes MOTH to provide masks compared to the classical workflow of loading pre-tiled image masks.

We utilized a real-world sample dataset of annotated mitosis, holding non-overlapping annotations, for this speed comparison and extracted tiles at a size of 375 pixels squared. We separated the initialization of the data structures from the generation of the masks, as it was only to be performed once.

The initialization process took approximately 2.17 s, compared to the 1.73 s needed to export the masks via Groovy in QuPath headless mode, which resembled the initialization in the classical workflow.

We afterward compared the generation of masks by MOTH for this sample, and the loading of tile masks from disks using OpenCV [14]. This was repeated 1000 times, and the results were averaged. MOTH needed an approximate time of 0.0075 s, while the loading process from disk takes took approximately 0.013 s, resulting in a performance boost when utilizing MOTH.

In our benchmark setup, we utilized SSD drives, which are inherently faster than the commonly used HDDs that typically offer larger storage capacities. Although large data projects involving whole slide images (WSIs) may tend to prefer HDDs due to their superior price-to-value ratio, our objective was to test MOTH against the faster storage variant. Given the design of HDD technology, we anticipate greater wear and tear on HDDs, attributed to their less favorable data block storage compared to SSDs. Although we compared MOTH to an on-disk method utilizing SSDs, MOTH is not significantly slower than storing the data on disk. Furthermore, MOTH offers greater flexibility and requires substantially less disk storage. Overall, these advantages make MOTH a favorable alternative to other on-disk methods. Despite this, storing tiles on on-disk remains widely accepted and utilized. Tools such as SlideTiler [15], which store all tiles locally on the hard drive, are particularly useful and convenient for single training runs. However, when different classes are needed to optimize the performance of an AI algorithm, for instance, when determining whether to include a specific histomorphology feature, multiple folders with tiles would be necessary. In contrast, MOTH can leverage the existing initialization to conduct various test scenarios without the need to create new tile folders for each run.

### 3.3. Comparison with Similar Tools

The existing Python packages HistomicTK and wsiprocess both provide utils to create tiled masks for whole slide image annotations in Python. Both packages offer similar functions to MOTH: extracting tiles in a flexible way by defining the area to be exported.

In comparison to HistomicTK, MOTH provides similar utils to export, import and stitch annotation masks. The difference lies in the supported annotation tool. HistomicTK. as part of the DSA. directly interacts with WSI and the annotation data stored in the DSA, whereas MOTH is implemented to interact directly with the WSI and annotation data stored in QuPath.

Wsiprocess, like MOTH, supports converting WSI and annotation data into tiles. While wsiprocess supports different tools, including QuPath, it requires the user to export annotations from QuPath via a Groovy script and manage WSI and annotation data files. MOTH exceeds wsiprocess by functioning as a direct tiling interface on the QuPath project. Therefore, the user does not have to manage WSI and annotation files, but can use the QuPath information directly.

### 3.4. Example Application of MOTH

**Figure 5:**
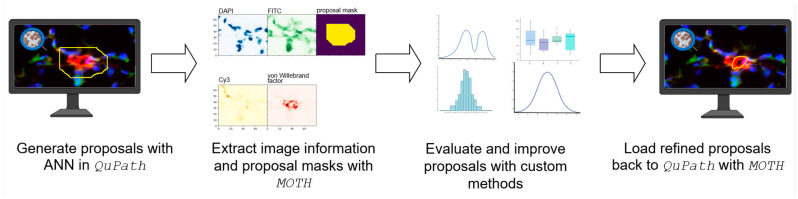
Real world example using MOTH. The proposals are generated via QuPath and extracted from the project via MOTH. The proposals are evaluated and improved via custom methods and loaded back into QuPath for visual inspection using MOTH.

To illustrate the utilization of MOTH, we present a case study of the processing of data using QuPath in a real-world context (Figure 5). We used whole mouse lung fluorescent microscopic images derived from tissue sections as an example application for MOTH, showing how to ingetrate MOTH into an existing AI workflow, and where it differs compared to a traditional pre-tiled workflow. The lungs were washed out to clear from blood, inflated through the trachea with a Tissue-Tek O.C.T (Sakura; Torrance, CA 90501, USA), and fixed overnight at 4 °C in 2% formalin, followed by overnight dehydration in 30% sucrose solution at 4 °C and long term storage at −80 °C. Five micron cryocuts were incubated for 10 min in an acetone:methanol mixture (1:1), washed with PBS, blocked with 3% bovine serum albumin, permeabilized by 0.5% Triton X-100, and processed using a ClickTech EdU Cell Proliferation Kit 488 (BaseClick, Bavaria, Germany) to visualize incorporated EdU in proliferating cells. Following the wash step, slides were incubated overnight at 4 °C with primary labeled antibodies against alpha smooth muscle actin, smooth muscle cell marker (Cy3 fluorophore, Sigma; St. Louis, MO 63178, USA), von Willebrand factor, endothelial cell marker (CF633 labelled antibody, Dako, Santa Clara, CA, USA), and nuclear counterstain (DAPI). Stained slides were imaged using a VS200 research slide scanner (Olympus; 20355 Hamburg, Germany) and digitalized for image analysis. The multi-channel fluorescent images contained information about specific morphological structures, which are were recognized in a first pass by an Artificial artificial Neural neural Network network (ANN) inside QuPath to generate proposals. ANN-mediated detection of specific morphological features in many cases recognizes either too small or too large segments, which might hinder precise morphometric analysis of identified features.

To characterize the proposals and subsequently enhance the corresponding quality, a more advanced method employed in a Python-based framework will be used. Individual proposals for annotations will be subjected to (1) validation of the proposals (i.e., identification and removal of false positive detections), (2) morphometric analysis, and (3) different image manipulation methods to refine the segment borders. The proposals are used in their discretized image mask format, and the corresponding image region is used as a starting point for the refinement to guarantee a more precise representation of the actual morphological features.

In the traditional Groovy-based workflow, data export and the Python backend for refinement would be decoupled. The mask proposals, as the starting point for the refinement, would be exported using Groovy scripting, storing the masks on the disk. The Python backend then requests the image/mask pairs from the disk and reads them. With the image/mask pairs now available, refining steps can take place by providing a new image mask. A visual inspection of the refined results in QuPath, and therefore of multiple regions at once, is impossible without additional processing steps.

In contrast, the MOTH workflow would skip the exporting script. Instead of reading the image/mask pairs from disk, the Python backend requests image/mask pairs directly via MOTH as an interface. The refinement steps do not change, since we provide the same input as in the traditional workflow. Importantly, after the refinement, MOTH offers us the possibility to view our refinements of multiple regions at once in QuPath by recombining multiple refined image masks and writing them into QuPath projects as polygons. This example shows that MOTH can be easily integrated into existing AI-driven workflows where image/mask pairs are utilized. Only I/O operations of the corresponding algorithms are affected, changing from a file-reading approach to request the regions via MOTH interface.

## 4. Conclusions

We presented here our Python package, called MOTH, for the on-the-fly tiling of annotated whole slide image tiles from QuPath projects, that can directly be forwarded to an AI algorithm, like U-net [16]. The results from the individual AI algorithm can then conveniently be transferred back to QuPath for visual inspection, using MOTH. The appended AI algorithm can be defined by the users for maximum flexibility. With our method, it is possible to use the annotated QuPath datasets highly flexible with different AI approaches and setups in a highly scalable and highly flexible manner. The on-the-fly transfer enables flexible tiling with changing overlaps, sizes, and positions to evaluate models with diverse input data. The automatic return of the results to the QuPath project after the analysis enables users to directly visually inspect the AI outcome, as a necessary step towards a complete digital pathology workflow. With our solution presented here, we are strengthening the open-source sector and helping to utilize or develop alternatives to purchased AI products [17,18,19,20].

## Figures and Tables

**Table 1:** Resulting mean and standard deviation for both compared datasets and compared export methods.

		MOTH IoU	Groovy IoU	MOTH HD [Pixel]	Groovy HD [Pixel]
artificial	mean	0.977994	0.955206	0.687514	1.365606
	std	0.017148	0.034553	0.020611	0.038121
mitosis	mean	0.981593	0.979179	0.9786	1.1322
	std	0.096959	0.096865	3.5592	3.5534

**Table 2:** IoU and HD of the well-defined examples.

Geometry	MOTH IoU	Groovy IoU	MOTH HD [Pixel]	Groovy HD [Pixel]
Small square	1.0	1.0	0.0	0.0
Offset square	0.6807	0.6807	0.7071	0.7071
Simple circle	0.852	0.6487	0.5851	1.0
Offset circle	0.8218	0.6693	0.6812	0.9841

## Data Availability

https://github.com/Neuropathology-Giessen/MOTH (accessed on 14 November 2024).
